# International Standards for Neurological Classification of Spinal Cord Injury: factors influencing the frequency, completion and accuracy of documentation of neurology for patients with traumatic spinal cord injuries

**DOI:** 10.1007/s00590-019-02502-7

**Published:** 2019-07-19

**Authors:** Temidayo Osunronbi, Himanshu Sharma

**Affiliations:** 1 Faculty of Medicine and Dentistry, University of Plymouth, Plymouth, UK; 2 Department of Physiology, Anatomy, and Genetics, University of Oxford, Oxford, UK; 3 Plymouth Spinal Services, South West Neurosurgery Centre, University Hospitals Plymouth NHS Trust, Plymouth, UK

**Keywords:** Spinal cord injuries, Trauma, Documentation, Neurologic examination, Standards

## Abstract

**Introduction:**

We aim to evaluate the effects of injury-related factors and clinician training grades on the frequency, completion and accuracy of International Standards for Neurological Classification of Spinal Cord Injury (ISNCSCI) charts in a tertiary care neurosurgery unit.

**Materials and methods:**

We retrospectively analysed 96 ISNCSCI charts of 24 traumatic spinal cord-injured (SCI) patients and 26 controls (vertebral fracture but neurologically intact), written by 50 clinicians. Seven components of each ISNCSCI charts (motor scores, sensory scores, sensory levels, motor levels, neurological level of injury, SCI severity and AIS) were reviewed to evaluate the effect of injury factors and clinician grade on the completion and accuracy of the ISNCSCI components.

**Results:**

The ISNCSCI chart was used 1.9 times on average during admission. The number of ISNCSCI assessments was significant in those with isolated spinal injuries (*p* = 0.03). The overall completion and accuracy rates of the assessed ISNCSCI chart components were 39% and 78.1%, respectively. Motor levels and AIS had the lowest completion rates. Motor levels and sensory levels had the lowest accuracy rates. The completion rate was higher in the charts of male patients, tetraplegic patients, and in patients with isolated spinal injuries. The junior clinicians had a significantly greater ISNCSCI chart completion rate than their seniors. However, the senior clinicians were more accurate in completing the ISNCSCI chart components.

**Conclusion:**

The quality of ISNCSCI documentation remained poor regardless of the clinician training grade and injury factors. Clinicians should be educated on the ISNCSCI protocol and the importance of adequate documentation.

**Electronic supplementary material:**

The online version of this article (10.1007/s00590-019-02502-7) contains supplementary material, which is available to authorized users.

## Introduction

The American Spinal Injury Association (ASIA) and International Spinal Cord Society (ISCoS) International Standards for Neurological Classification of Spinal Cord Injury (ISNCSCI) is the international communication tool used to determine the severity and level of the injury in patients with spinal cord injuries (SCI) [1–3]. The ISNCSCI involves a physical examination and the documentation of total motor and sensory scores, motor and sensory levels as well as a single neurological level of injury, an ASIA Impairment Scale, and severity of injury (complete/incomplete) [2, 4]. Clinicians can use these parameters from ISNCSCI to provide prognostic information such as the ability to walk, to patients and their families [4, 5]. Furthermore, the ISNCSCI helps to standardise practice and facilitate the evaluation of neurological symptoms to benefit patient care and aid research activities [1–4].

Patients with traumatic SCI initially spend between 1 and 12 weeks in an acute hospital bed [6–11]. In this period, documentation is usually performed by junior doctors who have variable training on neurological assessment. Several studies have shown that medical records by junior doctors were incomplete and lacked essential data on physical examination of the patients [12–15]. Previous studies conducted in the artificial settings of ISNCSCI training courses and clinical trials have revealed challenges in achieving accuracy in the completion of ISNCSCI charts [2, 3, 16, 17]. To our knowledge, no study has evaluated ISNCSCI chart completion and accuracy of documentation in a busy day-to-day clinical setting.

The aim of this study was to evaluate the effects of patient demographics, injury-related factors and clinician training grades on the frequency, completion and accuracy of ISNCSCI documentation for patients with traumatic SCI in a neurosurgery unit.

## Materials and methods

### Study design and setting

In this retrospective study, we evaluated 96 ISNCSCI charts of 24 traumatic spinal cord-injured (SCI) patients and 26 controls (vertebral fracture but no SCI), written by 50 clinicians in the neurosurgical unit of a regional trauma centre in the UK. The ISNCSCI charts were exclusively documented on paper. Only patients admitted from years 2012 to 2017 were included. Paediatric patients were excluded as this unit only treats adult patients.

In this hospital, the ISNCSCI is utilised predominantly clinically to diagnose SCI and monitor the patient’s neurology during admission. Clinical examination and neurophysiology studies are often carried out to differentiate SCI from non-SCI conditions such as traumatic plexopathy or neuropathy. Clinicians in the department are regularly advised to perform ISNCSCI within 24 h of the time of admission, immediate post-operative period and afterwards with any change in neurology identified at ward rounds. The senior clinicians (registrars and above) do a practical demonstration of the ISNCSCI examination and documentation to the junior doctors when they start the placement. The teaching is formalised but not standardised. In the present study, the clinicians involved in completing the ISNCSCI charts include two physiotherapists, two Specialist Registrars (SpR), seventeen Specialist/Core Trainees (ST/CT), eight Senior House Officers (SHO), ten Foundation Year 2 doctors (FY2), eight Foundation Year 1 doctors (FY1) and three final-year medical students.

The patient demographics considered in this study include age and gender. The injury-related factors considered are SCI severity (complete/incomplete), type of injury (tetraplegia/paraplegia), site of bony spine injury (cervical/thoracolumbar), non-spinal injury (including head injury (skull fracture, extradural and subdural haematoma), limb fracture, chest injury, abdominal injury, pelvic injury), spinal fusion surgery post-SCI (yes or no) and admission to intensive treatment unit (yes or no).

### Evaluation of completion and accuracy of the ISNCSCI charts

Determination of completion and accuracy of the components of the ISNCSCI charts was performed independently by the authors. The first author is a medical student that underwent intensive training on ISNCSCI examination and classifications using the published literature [18, 19] and the ASIA learning centre online training programme, InSTeP: International Standards e-Training program [1]. Self-study method has been reported to be an effective method of training medical students on ISNCSCI [20]. The senior author is a consultant spine surgeon since 2012 with years of experience of using ISNCSCI examinations and classifications. Both authors independently agreed on the accuracy of all components of the charts. The classifications by the authors were further validated through the European Multicenter Study about Spinal Cord Injury (EMSCI) online calculator [2].

The following components of the ISNCSCI chart were evaluated for completion and accuracy: motor scores (upper extremity motor score and lower extremity motor score), sensory scores (light touch total and pin prick total), sensory levels (right and left), motor levels (right and left), neurological level of injury (NLI), severity of the injury (complete or incomplete) and the ASIA Impairment Scale (AIS). Different versions of the ISNCSCI chart (2006 and 2015 versions) with different definitions of Zone of Partial Preservation (ZPP) were found in this study [21]. Moreover, there has been a recent (2019) update on the definition of ZPP, to include incomplete injuries. These recent changes to the definition of ZPP led to the omission of ZPP evaluation in the present study.

In evaluating the completion of the components of each ISNCSCI chart, a component is awarded a score of one if filled in and zero if blank (even if the component is non-determinable). For components with subcomponents (that is motor scores, sensory scores, sensory levels and motor levels), scores of two, one and zero are awarded if two, one or none of the subcomponents are filled in, respectively. In this study, there were 62 old versions (2006 version) ISNCSCI charts in which the NLI determination was not listed. The remaining 34 charts were the 2015 version where NLI determination was listed. Therefore, across the 96 ISNCSCI charts, a total of 994 components were assessed for completeness.

The completed components were subsequently assessed for accuracy. A completed component (or subcomponent, where applicable) was awarded a score of one if accurate or zero if inaccurate. Due to the retrospective nature of this study, the quality of ASIA examination could not be assessed. Thus, this study assumes that the dermatome, myotome and anorectal examinations were conducted appropriately by the clinicians. Consequently, accuracy is defined as the correct classification of the assessed ISNCSCI chart components based on the values documented by clinicians in the myotome, dermatome and anorectal examinations.

### Statistical analysis

Statistical analysis was performed on GraphPad Prism 8 (Windows). Two-tailed Mann–Whitney *U* test was used to evaluate the effects of patient demographics and injury-related factors on the frequency of ISNCSCI documentation. Two-tailed Fisher exact test was used to evaluate the effects of patient demographics, injury-related factors and clinician training grades on the completion and accuracy of the assessed components of the ISNCSCI chart. Two-tailed Spearman’s rank correlation coefficient was used to investigate the relationship between the completion rate and determinable rate of the assessed ISNCSCI chart. A *p* value < 0.05 was considered statistically significant.

## Results

There were 35 male (70%) and 15 female (30%) patients. The mean age was 56.1 years (range 20–93 years). Fifteen patients had tetraplegia, and nine had paraplegia. None of the patients had concomitant plexopathy, pre-injury neuropathy or multi-level SCI. The mean length of hospital stay was 18.5 days (range 4–67 days).

### The effect of patient demographics and injury-related factors on the frequency of ISNCSCI documentation

The mean number of ISNCSCI assessments was 1.9 per patient (range 0–10). In 23 patients (46%), there was no ISNCSCI assessment during hospital stay. The number of ISNCSCI assessments was higher in patients with SCI than in those who had no SCI on admission (*p* < 0.001). The number of ISNCSCI assessments was greater in patients with isolated spinal injuries than those that sustained additional non-spinal injuries (*p* = 0.03). No other significant associations were found between patient demographics/injury-related factors and the frequency of ISNCSCI assessment (Table 1).

**Table 1:** The effect of patient demographics and injury-related factors on the frequency of ISNCSCI documentation

Patient characteristics (*n* = 50)	Mean number of charts (per patient)	*p* values*
Gender		
Male (*n* = 35)	1.5	0.52
Female (*n* = 15)	2.8	
Age (years)		
Under 60 (*n* = 27)	2.1	0.85
60 and over (*n* = 23)	1.7	
Neurology on admission		
No SCI (*n* = 26)	0.4	< 0.001**
SCI (*n* = 24)	3.7	
SCI severity		
Complete (*n* = 5)	6	0.07
Incomplete (*n* = 19)	3	
Type of injury		
Tetraplegia (*n* = 15)	3.7	0.95
Paraplegia (*n* = 9)	3.4	
Site of bony spine injury		
Cervical (*n* = 26)	2.4	0.07
Thoracolumbar (*n* = 24)	1.4	
Non-spinal injuries		
Present (*n* = 25)	1	0.03**
Absent (*n* = 25)	2.8	
Management		
Spinal fusion surgery (*n* = 23)	2	0.63
Conservative (*n* = 27)	1.9	
ITU admission		
Yes (*n* = 13)	2.8	0.19
No (*n* = 37)	1.6	

*p values were calculated based on Mann–Whitney *U* test. **Significant at 5% significance level

### Completion and accuracy rates

**Fig. 1:**
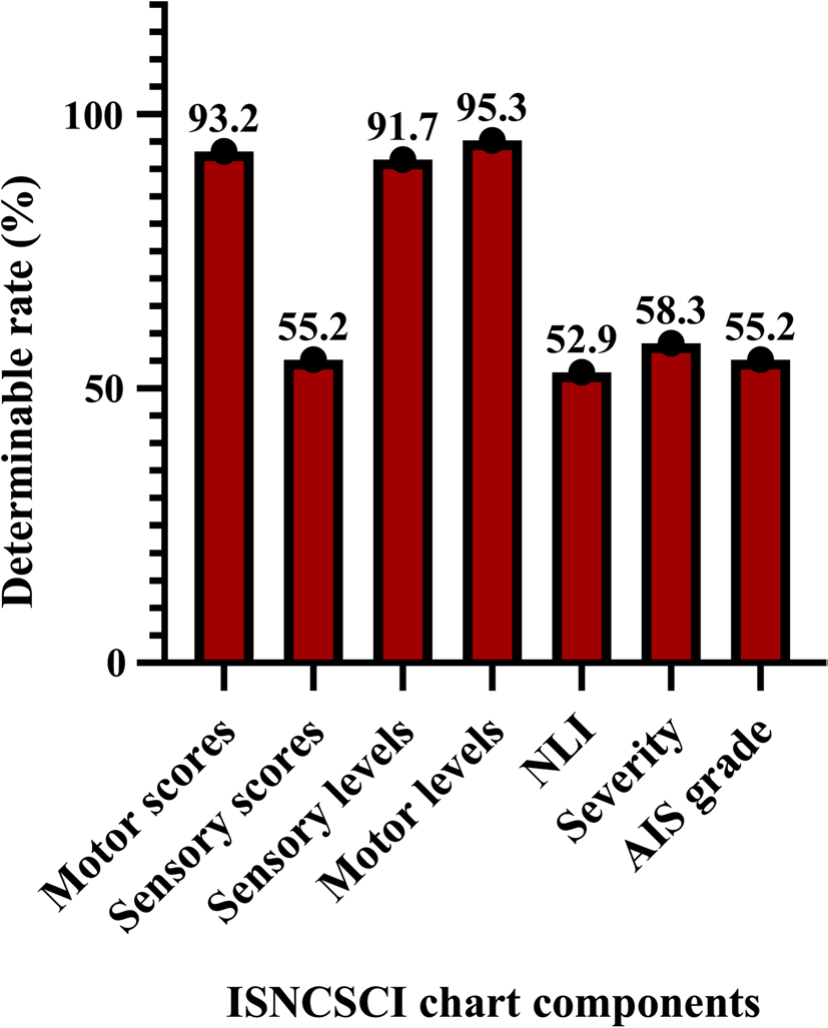
Determinable rates across the measured components of 96 ISNCSCI charts: motor scores (*n* = 192), sensory scores (*n* = 192), sensory levels (*n* = 192), motor levels (*n* = 192), NLI (*n* = 34), severity (*n* = 96), AIS (*n* = 96)

Myotome and dermatome values were blank and/or not tested in 10.4% and 38.5% of the 96 ISNCSCI charts, respectively. We noted that S4–S5 light touch, S4–S5 pin prick, voluntary anal contraction (VAC), and deep anal pressure (DAP) were blank and/or not tested in 42.7%, 42.7%, 51% and 51% of the 96 charts, respectively. All the aspects of ISNCSCI anorectal examination (VAC, DAP, S4–S5 light touch and pin prick) were blank and/or not tested in 33.3% of the ISNCSCI charts (*n* = 96). Sixty-one ISNCSCI charts had at least one non-tested myotome, dermatome or anorectal examination, but only 16.4% of the 61 charts commented on the reason for not testing the variables. The determinable rates of the assessed components in this study were measured based on the available values of the myotome, dermatome and anorectal examinations. The values of 77.6% of the 994 components could be determined. Motor scores (93.2%) and motor levels (95.3%) had the highest determinable rate, while the neurological level of injury (52.9%) had the lowest determinable rate (Fig. 1).

**Fig. 2:**
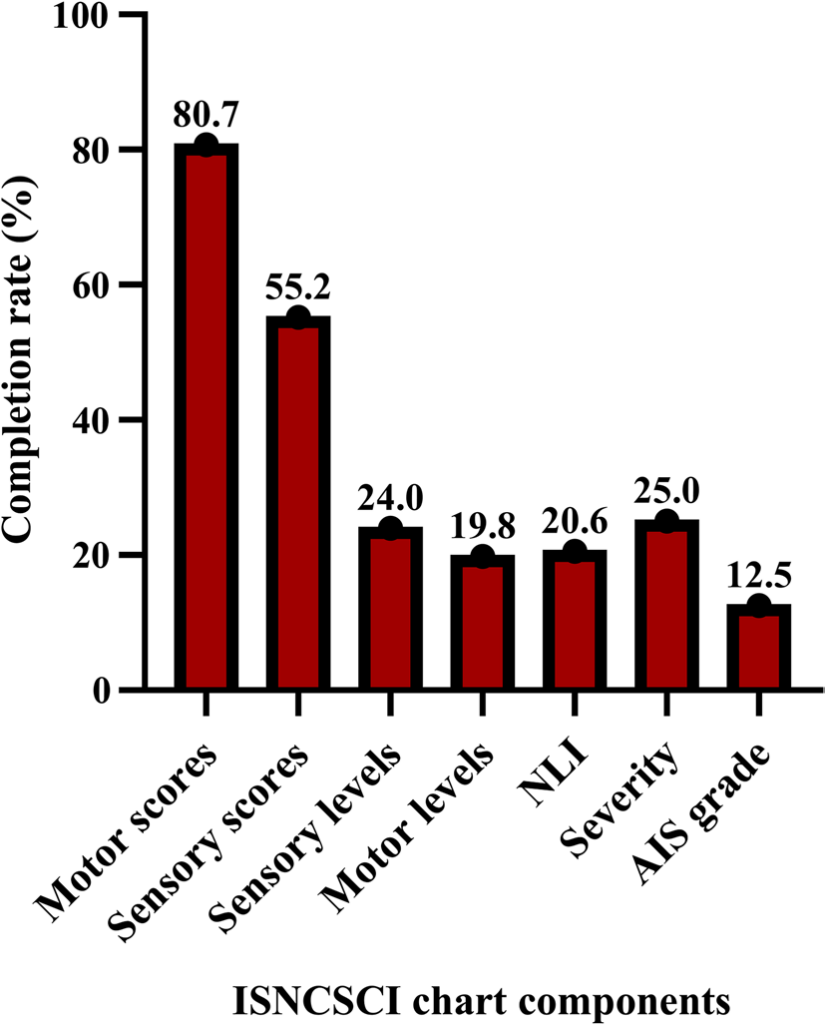
Completion rates across the measured components of 96 ISNCSCI charts: motor scores (*n* = 192), sensory scores (*n* = 192), sensory levels (*n* = 192), motor levels (*n* = 192), NLI (*n* = 34), severity (*n* = 96), AIS (*n* = 96)

Of the 994 measured components of the ISNCSCI charts, 39% were complete. Motor scores (80.7%) and sensory scores (55.2%) had the highest completion rate, while motor levels (19.8%) and AIS grades (12.5%) had the lowest completion rate (Fig. 2). There was no significant correlation between the determinable rate and the completion rate of the assessed ISNCSCI components (*r*_*s*_(5) = 0.16, *p* = 0.73).

**Fig. 3:**
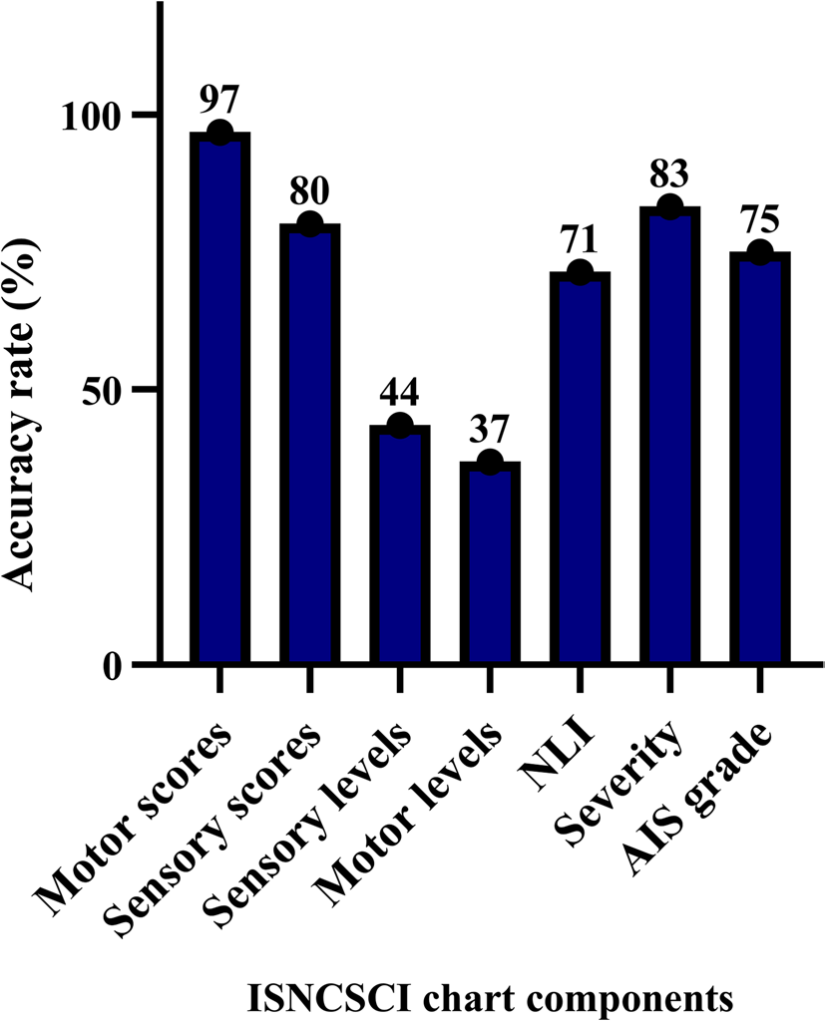
Accuracy rates across the measured completed components of 96 ISNCSCI charts: motor scores (*n* = 155), sensory scores (*n* = 106), sensory levels (*n* = 46), motor levels (*n* = 38), NLI (*n* = 7), severity (*n* = 24), AIS (*n* = 12)

Of the completed component (*n* = 388), 78.1% were accurate. Motor scores (96.8%) and SCI severity (83.3%) had the highest accuracy rate, while motor levels (36.8%) and sensory levels (43.5%) had the lowest accuracy rates (Fig. 3). The most common source of error in the determination of the motor levels was: violation of the ‘motor follows sensory rule’ in the levels without testable motor function (C1–C4, T2–L1) (23.8%) and violation of the rule that the key muscles above the determined motor level must be intact (18.4%). The most common source of error in the determination of the sensory levels was: some clinicians erroneously decided the level based on one of pin prick or light touch being intact instead of both (26%) and selection of the sensory level based on the first segment where sensation is 0 or 1 (21.8%). The common sources of errors in all the assessed components are described in Table 2.

**Table 2:** Error rates and types of errors in completed entries on ISNCSCI charts

ISNCSCI component	Errors in completed entries
Motor scores (*n* = 155)	∙ Calculated even though some myotomes were not tested: 3.2%
Sensory scores (*n* = 106)	∙ Calculated even though some dermatomes were not tested: 19.8%
Sensory levels (*n* = 46)	Error rate = 56.5% ∙ Unconventional use of numbers instead of spinal level: 8.7% ∙ Decided the level based on one of pin prick or light touch being intact instead of both (26%) The correct sensory level is one spinal level above: 13% The correct sensory level is six spinal levels above: 4.3% The correct sensory level is seven spinal levels above: 4.3% The correct sensory level is ten spinal levels above: 2.2% The correct sensory level is eleven spinal levels above: 2.2% ∙ Decided the level based on the first level where sensation is 0 or 1 (21.8%) The correct sensory level is one spinal level above: 10.9% The correct sensory level is two spinal levels above: 10.9%
Motor level (*n* = 38)	Error rate: 63.2% ∙ Unconventional use of numbers instead of spinal level: 10.5% ∙ The key muscles above the determined level are not intact (18.4%): The correct motor level is one spinal level above: 2.6% The correct motor level is two spinal levels above: 15.8% ∙ Assumed that the motor level is the most caudal segment where the key muscle is intact (graded as 5) (10.5%) The correct motor level is one spinal level above: 2.6% The correct motor level is two spinal levels above: 7.9% ∙ Violation of the ‘motor follows sensory’ rule in the levels without testable motor function (C1–4, T2–L1) (23.8%) The correct motor level is one spinal level below: 5.3% The correct motor level is four spinal levels below: 5.3% The correct motor level is five spinal levels below: 2.6% The correct motor level is six spinal levels below: 5.3% The correct motor level is ten spinal levels below: 5.3%
NLI (*n* = 7)	Error rate: 28.6% ∙ Due to wrong determination of sensory levels The correct NLI is one spinal level above: 14.3% The correct NLI is 9 spinal levels above: 14.3%
Complete/incomplete SCI (*n* = 23)	Error rate: 13% ∙ Classified as complete SCI even though there was sensation on deep anal pressure (4.3%) ∙ Classified as complete SCI even though S4–S5 dermatome is intact (4.3%) ∙ Classified as complete SCI when S4–S5 dermatome, voluntary anal contraction and deep anal pressure are not tested (4.3%)
AIS (*n* = 12)	Error rate: 25% ∙ Entered AIS A instead of AIS C: 8.3% ∙ Entered a number (total motor scores plus total sensory scores) instead of a grade: 8.3% ∙ Entered AIS E instead of non-applicable. The patient was neurologically intact. ASIA Impairment Scale does not apply to neurologically intact patients: 8.3%

‘*n*’ is the number of completed measured components

### The effect of patient demographics and injury-related factors on the completion of ISNCSCI documentation

The completion rate of the ISNCSCI chart components was higher in the charts of male patients compared to female patients (*p* = 0.005) and in the charts of patients with isolated spinal injuries compared to those that sustained additional non-spinal injuries (*p* = 0.001). The completion rate of the ISNCSCI chart components was greater in the charts of the control patients (vertebral fracture but no SCI) than those patients with SCI (*p* < 0.001), and in the charts of patients with tetraplegia than in those with paraplegia (*p* = 0.02). No other significant associations were found between patient demographics/injury-related factors and the completion of ISNCSCI documentation (Table 3).

**Table 3:** The effect of patient demographics and injury-related factors on the completion of components of the ISNCSCI chart

Patient characteristics	Proportion of ISNCSCI chart components completed (%)	*p* values*
Gender		
Male (*n* = 562)	42.8	0.005**
Female (*n* = 432)	34	
Age (years)		
Under 60 (*n* = 603)	38.6	0.79
60 and over (*n* = 391)	39.6	
Neurology on admission		
SCI present (*n* = 904)	36.3	< 0.001**
No SCI (*n* = 90)	66.7	
SCI severity		
Complete (*n* = 315)	39.1	0.22
Incomplete (*n* = 589)	34.8	
Type of injury		
Tetraplegia (*n* = 588)	39.1	0.02**
Paraplegia (*n* = 316)	31	
Site of bony injury		
Cervical (*n* = 476)	43.7	0.19
Thoracolumbar (*n* = 336)	39	
Non-spinal injuries		
Present (*n* = 264)	30.7	0.001**
Absent (*n* = 730)	42.1	
Management		
Spinal fusion surgery (*n* = 474)	42.4	0.051
Conservative (*n* = 520)	36.2	
ITU admission		
Yes (*n* = 370)	41.4	0.23
No (*n* = 624)	37.3	

*p values were calculated based on Fisher’s exact test. **Significant at 5% significance level

### The effect of clinician training grade on the completion and accuracy of ISNCSCI documentation

Ten ISNCSCI charts did not mention the examiner’s name on them; therefore, data from 86 charts were used in this analysis. For data analysis, we grouped clinicians into the categories described below (Table 4 and Table 5):

**Table 4:** The effect of clinician training grade on the completion of the measured components of the ISNCSCI chart

ISNCSCI charts by clinician training grade	Completion rate (%)						
	Motor scores^a^	Sensory scores^a^	Sensory levels^a^	Motor levels^a^	NLI^b^	Severity	AIS
Pre-registration (*n* = 25)	96	72	32	24	0	20	8
Post-registration (*n* = 61)	79.5	54.1	23	19.7	26.9	27.9	16.4
*p* values*	0.01**	0.04**	0.25	0.54	0.56	0.59	0.50
SHO and below (*n* = 50)	88	75	34	28	29.4	30	14
Above SHO (*n* = 36)	79.2	37.5	13.9	11.1	14.3	19.4	13.9
*p* values*	0.14	< 0.001**	0.003**	0.01**	0.41	0.32	>0.99
FY2/SHO (*n* = 25)	80	78	36	32	50	40	20
Above SHO (*n* = 36)	79.2	37.5	13.9	11.1	14.3	19.4	13.9
*p* values*	> 0.99	< 0.001**	0.008**	0.006**	0.09	0.09	0.73

*p values were calculated based on Fisher’s exact test. **Significant at 5% significance level

^a^Number of the measured components is double the number of charts

^b^Number of NLI for pre-registration group = 5; post-registratio*n* = 26; ‘SHO and below’ = 17; ‘above SHO’ = 14; FY2/SHO = 12

**Table 5:** The effect of clinician training grade on the accuracy of the completed measured components of the ISNCSCI chart

ISNCSCI charts by clinician training grade	Accuracy rate (%)						
	Motor scores	Sensory scores	Sensory levels	Motor levels	NLI	Severity	AIS
Pre-registration (*n*)	95.8 (48)	72.2 (36)	12.5 (16)	16.7 (12)	N/A (0)	100 (5)	100 (2)
Post-registration (*n*)	96.9 (97)	86.4 (66)	57.1 (28)	50 (24)	71.4 (7)	82.4 (17)	70 (10)
*p* values	> 0.99	0.11	0.005*	0.08	N/A	> 0.99	> 0.99
SHO and below (*n*)	97.7 (88)	78.7 (75)	41.2 (34)	42.9 (28)	100 (5)	86.7 (15)	100 (7)
Above SHO (*n*)	94.7 (57)	88.9 (27)	40 (10)	25 (8)	0 (2)	85.7 (7)	40 (5)
*p* values	0.38	0.39	> 0.99	0.44	0.048*	> 0.99	0.046*
FY2/SHO (*n*)	100 (40)	84.6 (39)	66.7 (18)	62.5 (16)	100 (5)	80 (10)	100 (5)
Above SHO (*n*)	94.7 (57)	88.9 (27)	40 (10)	25 (8)	0 (2)	85.7 (7)	40 (5)
*p* values	0.27	0.73	0.24	0.19	0.048*	> 0.99	0.17

*p values were calculated based on Fisher’s exact test. **Significant at 5% significance level. The number of measured components ‘*n*’ is in parentheses

- Pre-registration grades (medical student/FY1-Foundation year 1) versus post-registration grades (FY2—Foundation Year 2/SHO—Senior House Officer/CT—Core Trainee/ST—Speciality Trainee/SpR-Specialist Registrar/physiotherapist)

The overall completion rate of the pre-registration grades (46.7%, *n* = 255) was significantly greater (*p* = 0.04) than that of the post-registration group (39.2%, *n* = 636). The pre-registration grades were better at completing the motor scores, sensory scores, motor levels and sensory levels with significant differences in the completion rates of motor scores (*p* = 0.01) and sensory scores (*p* = 0.04).

The overall accuracy rate of the pre-registration grades (69.8%, *n* = 119) was significantly lesser (*p* = 0.01) than that of the post-registration group (82.3%, *n* = 249). The post-registration grade clinicians were more accurate than their juniors across the seven measured ISNCSCI components with a significant difference in the sensory levels (*p* = 0.005).

(b) ‘SHO and below’ grades (Medical student/FY1/FY2/SHO) vs ‘above SHO’ grades (CT/ST/SpR/physiotherapist)

The overall completion rate of the ‘SHO and below’ grades (48.9%, *n* = 517) was significantly greater (*p* < 0.001) than that of the ‘above SHO’ grades (31%, *n* = 374). The ‘SHO and below’ grade clinicians were better than their seniors at completing the seven measured components of the ISNCSCI charts, with significant differences in the completion of sensory scores (*p* < 0.001), sensory levels (*p* = 0.003) and motor levels (*p* = 0.01).

There was no significant difference (*p* = 0.77) between the overall accuracy rate of the ‘SHO and below’ grade (77.8%, *n* = 252) and the ‘above SHO’ grades (79.3%, *n* = 116). The ‘SHO and below’ grade clinicians were more accurate than their seniors across all the measured ISNCSCI components except sensory scores, with significant differences in NLI (*p* = 0.048) and AIS grades (*p* = 0.046).

(c) Post-registration subgroup analysis: FY2/SHO grades vs ‘above SHO’ grades (CT/ST/SpR/physiotherapist)

The overall completion rate of the ‘FY2/SHO’ grades (51.2%, *n* = 262) was significantly greater (*p* < 0.001) than that of the ‘above SHO’ grades (31%, *n* = 374). The ‘FY2/SHO’ grade clinicians were better than their seniors at completing the seven measured components of the ISNCSCI charts with significant differences in the completion of sensory scores (*p* < 0.001), sensory levels (*p* = 0.008) and motor levels (*p* = 0.006).

There was no significant difference (*p* = 0.25) between the overall accuracy rate of the ‘FY2 and SHO’ grade (85%, *n* = 133) and the ‘above SHO’ grades (79.3%, *n* = 116). The ‘SHO and below’ grade clinicians were more accurate than their seniors across all the measured ISNCSCI components except sensory scores and SCI severity, with a significant difference in NLI (*p* = 0.048).

## Discussion

In the initial catastrophic phase after injury, most patients want to know their long-term prognosis, especially the ability to walk [22]. The severity of the injury is the main prognostic factor for predicting ambulation outcomes after traumatic SCI [4, 5]. Therefore, it is impossible for clinicians to give patients the correct prognostic information if ISNCSCI assessment is not performed or documented accurately. Moreover, good documentation is essential in achieving good clinical practice, and more than ever, medical records are pivotal to medicolegal cases and litigations.

ISNCSCI assessments should be performed at least twice, ideally before and after treatment, to measure neurological changes during admission [23]. An earlier study by Lampart et al. investigated the frequency of assessment instruments for patients with SCI in a specialised SCI acute care and rehabilitation unit [23]. Lampart et al. and our study reveal that ISNCSCI charts were used 1.9 times on average, fulfilling the recommendation of performing ISNCSCI assessments at least twice. However, both studies show inconsistencies in the use of ISNCSCI charts, with 46% of our patients having no ISNCSCI charts in their records. In our study, the number of ISNCSCI assessments was significantly greater in patients with isolated spinal injuries than those who also sustained additional non-spinal injuries. This difference may be due to the increased likelihood of patients with non-spinal injuries spending some time in a non-neurosurgical ward during their hospital stay.

Our study revealed that clinicians were more likely to perform ISNCSCI assessments in patients that had SCI than those that had vertebral fracture but no SCI on admission. However, caution must be taken as a patient’s neurological status may change drastically during admission if they had inherently unstable spinal trauma or spontaneously reduced dislocated spine. In the SCI cohort, the number of ISNCSCI assessments did not significantly differ between those with complete lesions and those with incomplete lesions. Also, there was no difference in the number of ISNCSCI assessments between patients with tetraplegia and those with paraplegia. This contrasts with the study by Lampart et al. in which the frequency of ISNCSCI assessment was higher in patients with paraplegia than in those with tetraplegia [23]. To improve the consistency in the use of ISNCSCI charts, we suggest that for each patient, at least two ISNCSCI charts should be completed weekly. Due to time constraints, daily ISNCSCI is not feasible. While if ISNCSCI is done once a week, alterations in the neurology of the patient might be missed. If there is a rapid change in the neurological status of the patient, then additional ISNCSCI documentation should be conducted.

Non-tested components in ISNCSCI charts are reported to occur in up to 9% of cases in the literature [19]. In the present study, 63.5% of the ISNCSCI charts had at least one non-tested component (myotome, dermatome or anorectal examinations). We found that 39% of the 994 measured components on the ISNCSCI charts were complete. The completion rate was higher in the charts of patients without SCI than those with SCI, and this could be because it is less difficult and less time-consuming to document examination findings and classifications in the ISNCSCI chart when there are no neurological impairments. Interestingly, it was noted that the completion rate was greater in the charts of patients with isolated spinal injuries compared to those who sustained additional non-spinal injuries. It is possible that the presence of additional injuries such as limb fracture and its subsequent treatment causes some dermatomes and myotomes to be non-testable, leading to the non-completion of the measured ISNCSCI components. Motor scores and sensory scores cannot be generated if a myotome or dermatome is non-testable, respectively [1, 18, 19]. However, it is possible to determine the SCI severity and AIS grade, if the anorectal examination is performed (including the sacral segments) and if the non-tested segment does not make a difference in determining the AIS grade [19]. Although there was no correlation between the determinable rate and completion rate of components in this study, it is important to educate clinicians on the caveats in the cases of non-testable segments.

The accuracy rate of the completed components in the study was 78.1%. In earlier studies that evaluated fully documented ISNCSCI charts only, the pre-training accuracy of clinicians ranged from 25.5 to 73% [3, 16, 17]. On evaluating the completed entries only, the least accurate components were motor levels (36.8%) and sensory levels (43.5%). Previous studies identified the determination of motor level as the most challenging step [2, 3, 16, 17]. This has serious implications for prognostic information, as correct classification of AIS grades and NLI is dependent on the correct classification of the motor and sensory levels. In our study, most of the errors in the sensory levels were due to the clinician classifying the level based on one of pin prick or light touch being intact. This does not follow the ISNCSCI protocol which defines the sensory level as the most caudal, intact dermatome for both pin prick and light touch sensation. Most of the errors in the motor levels were because of violation of the ‘motor follows sensory’ rule in the levels without testable motor function (C1–4, T2–L1). Another common source of error in motor level determination was that the clinician did not follow the rule that all rostral key muscles above the level must be intact (MRC grade-5/5). These findings emphasise the need for clinicians to practise the rules for sensory and motor levels classifications in ISNCSCI training programmes.

Interestingly, the clinician training level had different effects on the completion rate and accuracy rates of the ISNCSCI components. This study showed that junior clinicians have a greater ISNCSCI chart completion rate than their seniors. This finding suggests that the hospital environment may have a negative effect on the clinician’s attitude towards ISNCSCI completion. The ISNCSCI chart is an elaborate form which takes reasonable time to complete, and therefore, there could be a tendency of disinterest in higher grades of junior doctors properly completing such tasks. Conversely, like an earlier study conducted in a training course setting [17], this present study showed that senior clinicians have a greater ISNCSCI chart accuracy rate than their juniors.

The inadequacies and inaccuracy in documentation found in the present study may be a consequence of lack of the essential knowledge or skills required for ISNCSCI documentation, negligence or lack of understanding of the importance of adequate documentation [3, 12–17]. The low completion and accuracy rates raise some questions on whether ISNCSCI should be used at all in an acute care clinical environment, if no quality control is installed and no structured ISNCSCI training is mandatory. However, ISNCSCI has two parts: the clinical examination of dermatomes and myotomes and determination and classification of the levels and the severity including the AIS. It might be that the clinical examination part already provides enough information for experienced neurosurgeons, whereas the classification part is more interesting for experts in field of spinal cord medicine.

To address the problematic pattern of ISNCSCI documentation in the acute care setting, clinicians should receive formal education on the ISNCSCI protocol and the importance of adequate documentation for patient safety, research purposes and medicolegal safety [13, 16, 17, 20]. Currently, the more senior clinicians in the department teach the more junior doctors how to perform ISNCSCI examination and documentation. However, the accuracy rate of the senior clinicians in this study is unacceptable and may have led to the lower accuracy rates in the more junior clinicians. Thus, we recommend that ISNCSCI training should be standardised and be handled by ISNCSCI experts, in addition to self-study using the published literature [18, 19] and the ASIA learning centre online training programme, InSTeP: International Standards e-Training program [1]. The senior clinicians can, however, play an important role in improving ISNCSCI documentation by showing commitment to adequate documentation and encouraging junior doctors to follow the rules. Regular audits may also encourage clinicians to maintain complete and accurate neurological documentation [13]. Utilising ISNCSCI calculators can reduce classification errors and may help clinicians with simple but time-consuming tasks such as obtaining the sensory and motor scores [2, 24]. However, clinicians should not rely exclusively on the ISNCSCI calculators, as human experts may be better than computational algorithms at dealing with complex cases of ISNCSCI classifications such as the presence of non-SCI conditions, and multi-level SCI [2].

Our study investigated the pattern of frequency, completion and accuracy of ISNCSCI charts in a day-to-day clinical setting. The major limitation of this study is the small sample size and single centre-based approach. A large multi-centre study involving other national and international hospitals and utilising the British Spine Registry database can provide a greater insight into the magnitude of problems regarding ISNCSCI documentation pattern. As there is very little data on the neurological outcomes of traumatic SCI on the registry, the proposed future study could be done prospectively. Furthermore, we could not verify the specific reasons why clinicians do not follow a comprehensive approach to ISNCSCI documentation as there could be element of educational need, lack of time, lack of interest or lack of guidelines on the frequency of neuro-charting. Future research could use questionnaires and focus-group discussions to investigate the specific reasons why clinicians do not document ISNCSCI charts comprehensively. Understanding why this occurs is necessary to design a proper approach for solutions.

## Conclusion

The dataset from this retrospective study suggests that gender, absence or presence of non-spinal injuries, injury type (tetraplegia/paraplegia) and clinician training grade may influence ISNCSCI documentation patterns for patients with traumatic SCI admitted to an acute neurosurgical unit. The quality of ISNCSCI documentation remained poor regardless of the patient or clinical factors and clinician training grade. We outlined potential reasons and solutions for the low inadequacy and inaccurate completion of ISNCSCI charts in the hospital environment. The proposed reasons for the low quality of ISNCSCI documentation include negligence, lack of knowledge, and the time-consuming nature of the ISNCSCI examination and documentation. Future research should investigate these reasons to design solutions to improve the quality of documentation. In addition, clinicians should be educated on the ISNCSCI protocol, especially the determination of motor and sensory levels, and the importance of adequate documentation.

## Electronic supplementary material

Below is the link to the electronic supplementary material.

**Supplementary file**: Raw data of patient demographics and completion of components of ISNCSCI chart (XLSX 26 kb)
